# Plant adaptation to drought stress

**DOI:** 10.12688/f1000research.7678.1

**Published:** 2016-06-30

**Authors:** Supratim Basu, Venkategowda Ramegowda, Anuj Kumar, Andy Pereira

**Affiliations:** 1 Crop, Soil, and Environmental Sciences, University of Arkansas, Fayetteville, Arkansas, 72701, USA

**Keywords:** Adaptation, Drought tolerance, drought resistance, grain yield, rice, photosynthesis

## Abstract

Plants in their natural habitats adapt to drought stress in the environment through a variety of mechanisms, ranging from transient responses to low soil moisture to major survival mechanisms of escape by early flowering in absence of seasonal rainfall. However, crop plants selected by humans to yield products such as grain, vegetable, or fruit in favorable environments with high inputs of water and fertilizer are expected to yield an economic product in response to inputs. Crop plants selected for their economic yield need to survive drought stress through mechanisms that maintain crop yield. Studies on model plants for their survival under stress do not, therefore, always translate to yield of crop plants under stress, and different aspects of drought stress response need to be emphasized. The crop plant model rice ( *Oryza sativa*) is used here as an example to highlight mechanisms and genes for adaptation of crop plants to drought stress.

## Introduction

Drought stress is the most prevalent environmental factor limiting crop productivity ^1^, and global climate change is increasing the frequency of severe drought conditions ^2^. The sheer diversity of plant species grown across climatic regions that include extreme dry conditions suggests that, in nature, plants have evolved to endure drought stress with an array of morphological, physiological, and biochemical adaptations ^3^. ‘Drought resistance’ (DR) is a broader term applied to plant species with adaptive features that enable them to escape, avoid, or tolerate drought stress ^4^. ‘Drought escape’ is the ability of a plant species to complete its life cycle before the onset of drought. Thereby, plants do not experience drought stress, as they are able to modulate their vegetative and reproductive growth according to water availability, essentially through two different mechanisms: rapid phenological development and developmental plasticity ^5^. Rapid phenological development involves rapid plant growth, producing a minimal number of seeds before the soil water depletes, and these plants are considered not to have any special morphological, physiological, or biochemical adaptations. Plants with mechanisms of developmental plasticity show little growth during the dry season, with very few flowers and seeds, but in wet seasons they grow indeterminately, producing a large amount of seed. ‘Drought avoidance’ is the ability of plants to maintain (relatively) higher tissue water content despite reduced water content in the soil ^4^. This is achieved through a variety of adaptive traits involving the minimization of water loss (water savers) and optimization of water uptake (water spenders). Water spenders achieve higher tissue water status by maintaining the water uptake through increased rooting, hydraulic conductance, etc. under drought stress. In contrast, water savers use water effectively through reduced loss of water by reducing transpiration, transpiration area, radiation absorption, etc. under drought stress. ‘Drought tolerance’ (DT) is the ability of plants to endure low tissue water content through adaptive traits. These adaptive traits involve maintenance of cell turgor through osmotic adjustment and cellular elasticity, and increasing protoplasmic resistance ^6^.

Improvement of yield and maintaining yield stability of crops, under normal as well as drought stress conditions, is essential for the food security of the growing global population. It is difficult to resolve the role of different components of DR in the stability of the crop yield as the major objective. However, there exist a variety of different mechanisms for drought escape, avoidance, or tolerance in natural populations that can improve DR and maintain grain yield in crop plants. In nature, extreme DR is found in resurrection plants ^7, 8^ which possess strong drought escape mechanisms. Resurrection plants can be exposed to severe drought for months, extending up to years, forcing them to optimize their growth for survival, but not for seed production, in the long term ^9^. Therefore, the DR mechanisms that enable plants to merely survive longer lead to subsistence yield, which is much lower than that which is observed under normal conditions. Crop plants, on the other hand, are grown by humans in environments under conditions for high agricultural production and will be exposed to only a random short-term drought stress of days to weeks, from which they must quickly respond to limit the damage caused by short-term drought stress while they continue to grow and yield in the stressful environments. Therefore, bringing in the drought adaptive mechanisms from plants adapted to grow in extreme dry conditions may not be a feasible option, as it may result in growth and/or yield penalty in crop plants under drought as well as normal conditions.

Although plant survival is very critical in the early growth stages, the mechanisms have little relevance to increasing grain yield directly. The emphasis to improve DR of crop plants should therefore be based on stability of yield components and not on plant survival alone. So far, most of the efforts to improve grain yield under drought stress were focused on secondary traits such as root architecture, leaf water potential, osmotic adjustment, and relative water content at the vegetative stage, which are often not highly correlated with grain yield ^10, 11^. Looking forward in crops, the effective drought improvement approach should be selection for yield and its component traits under reproductive-stage drought stress ^12^. Additionally, little importance has also been given to simultaneous improvement of grain yield under normal and drought conditions. Selection for DT has been suggested to have a yield drag under normal conditions. It has been proposed that the yield potential of crop plants should be simultaneously selected for under favorable and environmental stress conditions, as there is a positive correlation between yield potential under normal and drought stress conditions ^13^. Combining high yield potential under normal conditions with good yield under drought stress is the ideal trait. Identification of mechanisms, traits, and genes regulating yield under drought stress that are free from yield drag under normal conditions should be the focus. For example, regulation of yield under normal as well as drought stress conditions has been shown for three NAC family transcription factors (TFs). Transgenic plants expressing *OsNAC5*, *OsNAC9*, and *OsNAC10* TFs showed an increase in grain yield of 5-26% under normal conditions ^14– 16^. Nevertheless, in these studies, yield under normal conditions has been overlooked with more emphasis given to yield under drought stress. Two of our recent studies show the potential of simultaneously improving and stabilizing grain yield, both under normal as well as drought stress conditions, using two regulatory genes, namely *GUDK* and *HYR* in rice ^17, 18^. These studies indicate that it might be advantageous to identify mechanisms and genes for increasing grain yield that are also stable or maintained under drought stress conditions.

Despite the complexity of DR, tremendous progress has been made in understanding the drought-adaptive mechanisms of plants ^1, 19, 20^. Adaptation through DR mainly involves morpho-physiological alterations. These alterations in adaptive processes are controlled by molecular mechanisms that regulate the expression of genes ^21^. There exists a large diversity in drought adaptation within a crop species, as some genotypes are able to cope with drought better than others. Genotypes that differ in drought adaptive mechanisms serve as an important resource to study the variation in drought adaption in crop plants. This natural variation needs to be exploited to simultaneously improve DR and yields of cultivated varieties through better understanding of the underlying mechanisms and to aid in selection for these traits ^22^. In the following sections, we describe the widely known morpho-physiological processes and recent molecular advances in regulating these drought-adaptive processes leading to increased yield in crop plants.

## Photosynthesis

Drought stress is known to reduce photosynthesis by decreasing both leaf area and photosynthetic rate per unit leaf area. Reduced photosynthetic rate is mainly through stomatal closure or metabolic impairment ^23^. Continued photosynthetic light reactions during drought stress under limited intercellular CO _2_ concentration results in the accumulation of reduced photosynthetic electron transport components, which can potentially reduce molecular oxygen, resulting in the production of reactive oxygen species (ROS). ROS can cause severe damage to the photosynthetic apparatus ^24^. The adaptive responses that plants have developed to reduce drought-induced damage to photosynthesis include thermal dissipation of light energy, the xanthophyll cycle, the water-water cycle, and dissociation of the light-harvesting complexes from photosynthetic reaction centers ^25– 27^. The metabolic impairment during drought stress is mainly caused by changes in photosynthetic carbon metabolism ^24^. The biochemical efficiency of photosynthesis under drought stress mainly depends on ribulose-1,5-bisphosphate (RuBP) regeneration and the activity of ribulose-1,5-bisphosphate carboxylase/oxygenase (RuBisCO) ^28, 29^. Considerable progress has been made in improving the stomatal components for CO _2_ diffusion, photosynthetic light reaction, and metabolic changes, including the expression of photosynthesis-related genes to regulate photosynthesis under drought towards the improvement of grain yield ^30^.

The C4 pathway of carbon assimilation has been suggested to be the major adaptation of the C3 pathway to limit water loss, reduce photorespiration, and improve photosynthetic efficiency under drought stress ^31^. However, many important crops—including rice, wheat, soybean, and potato—use the C3 pathway of photosynthesis. Although the transfer of the C4 pathway into C3 crops is underway, so far its contribution to increased grain yield is very limited ^32^. Photosynthetic adaptation of plants to drought stress involves a complex interaction of hormones, ROS, sugars, and other metabolic events ^33^. Combinations of computational models, which integrate the physiological and metabolic processes with gene expression data, along with modern breeding and transgenic technologies hold promise in improving photosynthesis and hence crop yield under normal as well as drought stress conditions.

In recent studies, we used a rice gene regulatory network to identify a TF termed HYR (HIGHER YIELD RICE), which was highly associated with primary carbon metabolism ^17^, and on overexpression in rice enhanced photosynthesis under normal conditions as well as under drought and high temperature stress. HYR regulates several morpho-physiological processes leading to higher yield under normal and environmental stress conditions. Our study showed that HYR is a master regulator of photosynthesis, directly activating photosynthesis genes, cascades of TFs, and other downstream genes involved in photosynthetic carbon metabolism, resulting in improved yield.

## Hormonal regulation

**Figure 1.**
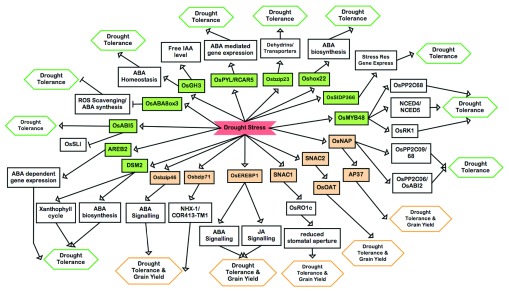
The abscisic acid (ABA)-dependent gene regulatory pathway in rice. This pathway is required for drought stress tolerance and grain yield under drought. The drought stress ABA-dependent signal is shown perceived directly by the regulatory genes described in the text, followed by transcriptional regulation of downstream genes and underlying stress response mechanisms. Genes regulating drought tolerance (DT) at the vegetative stage are shaded green, and genes regulating DT and grain yield under drought are shaded orange. The resulting phenotypes are represented for DT at the vegetative level only (green diamonds), or DT and grain yield (orange diamonds). The genes described here are *OsGH3* ^92^, *OsNAP, OsABI2* ^39^, *AP37* ^93^, *OsPP2C09*, *OsPP2C06* ^94^, *OsPYL*/ *RCAR5*, *OsSIDP366* ^95^, *OsMYB48* ^96^, *OsRK1* ^97^, *Oshox22* ^98^, *SNAC2* ^99, 100^, *OsOAT* ^101^, *OsbZIP23* ^102^, SNAC1 ^99^, OsEREBP1 ^103^, *OsbZIP71* ^104^, *OsbZIP46* ^105^, *OsABI5* ^106^, *DSM2* ^40^, *AREB2* ^107^, *OsSRO1c* ^108^, and *OsABA8OX3* ^109^.

Major phytohormones, such as abscisic acid (ABA), cytokinin (CK), gibberellic acid (GA), auxin, and ethylene, regulate diverse processes which enable plant adaptation to drought stress ^34^. Upon exposure of plants to drought stress, ABA is the major hormone synthesized in roots and translocated to leaves to initiate adaptation of plants to drought stress through stomatal closure and reduced plant growth ^35^. However, modulating the ABA-induced drought adaptation of plants for better yield remains a greater challenge because of the potential inadvertent reduction in carbon gain upon stomatal closure and ABA-induced senescence, especially if the drought occurs at the reproductive stage ^36^. There are ABA signaling genes, such as *OsNAP*, *OsNAC5*, and *DSM2*, which promote improved yield under reproductive drought ^37– 40^. These ABA-induced non-stomatal adaptations of plants under drought stress can be exploited to improve grain yield under reproductive drought ( Figure 1).

Under drought stress, CKs are known to delay premature leaf senescence and death, adaptive traits very useful for increasing grain yield. An increase in the endogenous levels of CK through expression of *isopentenyltransferase* ( *IPT*), a CK biosynthetic pathway gene, leads to stress adaptation by delaying drought-induced senescence and an increase in yield ^41, 42^. Generally, auxin has been shown to negatively regulate drought adaptation in plants. Decrease in indole-3-acetic acid (IAA) content was shown to be associated with up-regulation of genes encoding late embryogenesis abundant (LEA) proteins, leading to drought adaptation in plants ^43, 44^. Recently, the *DEEPER ROOTING 1* ( *DRO1*) gene determining a quantitative trait locus (QTL) controlling root growth angle was shown to be negatively regulated by auxin. Higher expression of *DRO1* in a shallow-rooting rice cultivar resulted in drought avoidance and high yield under drought ^45^. GA is suggested to positively regulate plant adaptation to drought stress. A rapid decline in levels of endogenous GA was observed in plants subjected to drought stress, resulting in growth inhibition ^46^. The role of GA in regulating grain yield of crop plants is thus an important area that can be further explored. Ethylene is a negative regulator of drought stress response by promoting leaf senescence and inhibiting root growth and development, shoot/leaf expansion, and photosynthesis ^47– 51^. Ethylene can also directly affect yield by increasing embryo and grain abortion and reducing the grain-filling rate ^52^. In addition to the major hormones, other hormones such as brassinosteroids, jasmonic acid (JA), salicylic acid (SA), and strigolactone also have an equally important role in plant growth and development. However, their function under drought stress is relatively less characterized. Tillering in rice has been suggested to be the outcome of an interaction among three hormones, CK, auxin, and strigolactone, with CK promoting branching and the other two inhibiting it ^53, 54^, suggesting that all hormones do not act in isolation but instead interact and modulate each other’s biosynthesis and responses. Therefore, the net outcome of the drought stress response is regulated by a balance between hormones that promote and those that inhibit the trait, rather than individual hormones.

## Transpiration and stomatal conductance

The immediate response of plants on being exposed to drought stress is stomatal closure. However, stomatal closure not only diminishes water loss through transpiration but also reduces CO _2_ and nutrient uptake, and hence alters metabolic pathways such as photosynthesis ^55^. Plants growing in dry areas have developed xeromorphic traits to reduce transpiration under drought stress. Reduction in transpiration under drought stress conditions can also be achieved through leaf shedding (i.e. deciduous species in drought) as well as decrease in leaf number, leaf size, and branching. Another adaptation to counter drought stress is sclerophylly, where plants form hard leaves that will not suffer permanent damage due to wilting and can be restored to full functionality when normal conditions resume ^56^. Recent research has shown that decreased stomatal conductance in response to drought stress is related not only to reduced expression of aquaporin genes but also to anatomical traits leading to reduction of chloroplast surface area exposed to intercellular space per unit leaf area ^57, 58^. Several other factors, including leaf developmental stage and light availability, are also known to interact with drought in modulating mesophyll and chloroplast differentiation, ultimately affecting conductance and photosynthetic capacity ^58^. Reduction in stomatal size and number on exposure to drought is another adaptation for survival under drought conditions. Previous studies have shown that while there is an increase in stomatal density under mild drought stress, there is a decrease during severe drought ^59^. Thus, all these adaptations in plants reduce the negative impacts of drought stress on photosynthesis and thereby have a positive effect on water use efficiency (WUE), which in turn will result in high yield potential and high yield ^60^. Such an adaptation was shown in rice by overexpression of the Arabidopsis AP2/ERF TF *HARDY* that improved WUE (the ratio of biomass produced to water used) by enhancing photosynthesis and reducing transpiration ^61^. The above reported traits therefore exemplify adaptive mechanisms in plants to survive under drought stress without loss of productivity or yield.

## Root morphology

In many agriculturally important crops, drought stress is perceived first by the root system, which continues to grow underneath the soil even though shoot growth is inhibited under these conditions ^62^. Although the growth of the primary root is not affected by drought stress, the growth of lateral roots is significantly reduced, mainly by suppression of the activation of the lateral root meristems ^63^. The Arabidopsis R2R3-type MYB TF *MYB96* has been shown to regulate activation of lateral root meristem through an ABA signaling cascade, with an activation-tagged mutant showing enhanced DR with reduced lateral root formation ^64^. The plant microRNA miR393 has also been shown to play a role in root-mediated adaptation to drought stress response through attenuation of auxin signaling ^65^. In addition to the lateral roots, the presence of small roots is also considered as an adaptive strategy to increase water uptake by providing more absorptive surface. Presence of specialized tissues like rhizodermis, with a thickened outer cell wall or suberized exodermis, or reduction in the number of cortical layers are considered an adaptive advantage for drought stress survival. Hydrotropism is another adaptive measure taken by plants to counter stress, where studies have shown that degradation of amyloplasts in the columella cells of plant roots on exposure to drought stress increases hydrotropism ^66, 67^. Hormonal cross-talk mediated by auxin, CK, GA, and ABA has been implicated as a potential chemical signal in response to water stress to modulate root system architecture ^68^.

The expression of enzymes related to root morphology (e.g. xyloglucan endotransglucosylase) is induced upon mild drought stress, while other structural proteins are down-regulated, which is strongly correlated with root growth and hence an augmentation in the surface area for water uptake. The alterations in the expression of these proteins correlate positively with lateral development that in turn also affects photosynthesis ^69^. More lateral root and root hair formation was found in lines possessing a QTL, qDTY12.1, only when under drought ^70^. Such traits, which are expressed only under drought stress, have higher potential to increase grain yield under drought. Moreover, it has also been shown that drought stress triggers a wide variety of anatomical traits expressed to different levels and patterns in different species and even in different cultivars within species ^71– 73^. For example, suberization and compaction of sclerenchyma layer cells were shown to decrease in rice under drought, which increases retention of water under drought stress ^71^.

## Osmotic adjustment

Osmotic adjustment (OA) is defined as a process of solute accumulation in dividing cells when the water potential is reduced, and thereby helps in maintaining the turgor ^74^. Cell enlargement and growth in plants is highly dependent on water availability and helps in maintaining the turgor. Turgor measurement in growing regions of plants, especially the leaves and stems, shows little or no reduction, though cell enlargement is inhibited during drought stress and is believed to be due to OA ^75, 76^. Under conditions of drought stress, OA has been implicated in maintaining stomatal conductance, photosynthesis, leaf water volume, and growth ^74, 77^. At times of drought stress, in addition to the reduction in water content, there are also other associated changes, such as increases in salt concentration and mechanical impedance ^78^. Inorganic cations, organic acids, carbohydrates, and free amino acids are the known predominant solutes that accumulate in response to water stress. Previous studies have shown that drought-resistant wheat varieties, with yield stability under drought stress, have a greater capacity for osmoregulation than less resistant varieties ^76^. The accumulation of compatible solutes such as proline and glycine betaine help in protecting the plants from detrimental effects of drought stress not only by OA but also by detoxification of ROS, protection of membrane integrity, and stabilization of enzymes or proteins ^79^. Enzymes such as betaine aldehyde dehydrogenase (BADH), pyrroline-5-carboxylate reductase (P5CR), and ornithine δ-aminotransferase (OAT) have been shown to play major roles in OA. Overexpression of Arabidopsis *EDT1/HDG11* was shown to increase DT of poplar and cotton through increased accumulation of solutes such as proline and soluble sugars and also increase the yield of cotton in the field ^80^. However, there are some plants in which sugars are the main osmolytes that play a significant role in OA, including sucrose, trehalose, glucose, and fructose. Previous studies have shown that overexpression of the *sucrose:fructan-6-fructosyltransferase* ( *6-SFT)* gene from *Psathyrostachys huashanica* in tobacco and the trehalose-6-phosphate phosphatase gene *OsTPP1* in rice confers abiotic stress tolerance ^81, 82^. Researchers have also identified a QTL for OA on chromosome 8 in rice that is homeologous with a segment of wheat chromosome 7 ^83^.

## Source-sink relationships

Source-sink relationships largely determine the grain yield of cereal crops, with developing grains being primary sinks while the top two leaves, the flag leaf in particular, are the primary source ^84, 85^. Drought stress affects the source-sink relationship by reducing the source strength, leading to yield reduction. Sufficient sugar supply through photosynthesis, transport, and conversion of sugars is regarded as the most critical component in determining the viability of reproductive organs in rice ^86– 88^. Drought stress dramatically affects pollen viability due to abnormal starch accumulation ^89^. Insufficient starch synthesis and arrested pollen development have been linked to reduced invertase activity under drought stress ^88^. In addition to invertases, active grain filling involves other key enzymes, such as sucrose synthase, ADP glucose pyrophosphorylase (AGPase), and starch synthase as well as starch branching and debranching enzymes, which are also affected by drought stress ^88^. Therefore, enhancing rice yield through source-sink relationships involves not only sugar metabolism but also the regulated mobilization of metabolic resources from source to sink tissue.

## Future research perspectives

Improving DR in crop plants is a challenge for plant breeders and crop physiologists, as it is a complex genetic trait with multiple pathways involved. Effective development of drought-resistant crop plants thus requires the pyramiding and interaction of many mechanisms, traits, and genes that are appropriate to individual crops and their growing environments. Success in this direction not only extends the growing area of crop plants but also achieves stable yield in drought-prone areas. Identifying genetic variation for DR is the first step towards development of drought-resistant crop plants. Such variation is often present in wild species and adapted genotypes that have evolved under natural selection and these are the best source of DR traits. Evaluation of these resources through an integrated phenotyping and genotyping approach under field conditions alongside identification of traits that are directly associated with yield is key to improving DR ^90^. Comparative ** omics analyses between the diverse germplasm could aid in bettering our understanding of the variety of crop adaptations to drought stress. In addition, analysis of specific genes focussed on increasing DR while stabilizing the yield is crucial for understanding the broad basis of complex traits such as DR ^91^. The development of high-yielding resilient crops ^74^ that maintain yield stability under drought and other environmental stresses due to climate change is also currently needed. Drought-resistant plants should combine a better root system, stomatal regulation, WUE, and hormonal balance while avoiding the negative effects on grain yield under both normal and drought stress conditions. Therefore, a holistic crop improvement strategy should involve the deployment of high crop yield potential and the utilization of a combination of morpho-physiological, biochemical, and anatomical adaptive responses to drought stress.
